# Supplementary material for: Active Forms of Chemerin Are Elevated in Human and Mouse Ovarian Carcinoma
Source: Biomedicines. 2025 Apr 18;13(4):991. doi: 10.3390/biomedicines13040991 (PMC12024974; doi:10.3390/biomedicines13040991)
Supplement: Supplementary file 1 [file biomedicines-13-00991-s001.zip › biomedicines-3527369-supplementary.pdf]

Table S1

Study participant	<25 BMI medications
001	aspirin
004	aspirin, lisinopril

Study participant	>40 BMI medications
100	amlodipine, atorvastatin, citalopram, empagliflozin, insulin, hydrochlorothiazide, lisinopril, metformin, omeprazole, vitamin D
101	citalopram, clonidine, gabapentin, melatonin, prazosin, vitamin D
103	atenolol, cyclobenzaprine, fluoxetine, gabapentin, glipizide, hydrochlorothiazide, insulin, indomethacin, losartan, rosuvastatin, tamsulosin
104	aspirin, bupropion, citalopram, gabapentin, ibuprofen, lurasidone, omeprazole, topiramate, vitamin D
105	atorvastatin, glipizide, insulin, lisinopril, pregabalin, saxagliptin, sertraline, sildenafil, tramadol, vitamin D
106	amphetamine/dextroamphetamine, aripiprazole, carvedilol, cyclobenzaprine, escitalopram, felodipine, furosemide, gabapentin, insulin, lisinopril, pantoprazole, prazosin, simvastatin, spironolactone, tramadol, trazodone, vitamin B, vitamin D
107	atorvastatin, cyanocobalamin, metformin, vitamin D

108	acyclovir, albuterol, atorvastatin, buspirone, cetirizine, cyclobenzaprine, fluoxetine, fluticasone, gabapentin, hydroxyzine, levothyroxine, liraglutide, multivitamins, vitamin D
110	buspirone, duloxetine, etodolac, gabapentin, omeprazole, propranolol, vicodin, multivitamins
111	calcium, lisinopril, omeprazole, multivitamins
113	amlodipine, divalproex, lisinopril, metformin, methocarbamol, metoprolol, pravastatin
114	bupropion, esomeprazole, hydroxazine, ranitidine
115	acyclovir, vitamin D
116	ambien, paxil, pantoprazole, ranitidine
117	albuterol, atorvastatin, diltiazem, ferrous sulfate, fluticasone, hydrochlorothiazide, losartan, metformin, ranitidine
118	diclofenac, triamcinolone
119	saxenda, spironolactone
120	lisinopril, omeprazole, tizanidine, vitamin D
121	amlodipine, atorvastatin, cyclobenzaprine, diclofenac, hydrocodone/acetaminophen, lamotrigine, loratadine, metoprolol, nortriptyline, omeprazole, pregabalin, multivitamins
123	duloxetine, ergocalciferol, gabapentin, hydroxyzine, omeprazole, prazosin, ranitidine, sumatriptan
150	lisinopril, metformin, motrin, vitamin D
151	calcium, cyanocobalamin, duloxetine, estradiol, levothyroxine, omeprazole, trazodone, vitamin D

Medications reported by participants.

Table S2

Study participant	Histology	Stage	Platinum status	Therapy agent or regimen
120	high-grade serous fallopian tube carcinoma	benign	sensitive	single-agent carboplatin C1/C2, C3 carboplatin/taxol, C4-6 carboplatin/abraxane
128	high-grade serous ovarian carcinoma	IIB	resistant	adjuvant carbo/taxol 6C, 4C carboplatin/doxil, ?C cisplatin/gemzar, 8C topotecan/avastin, niraparib maintenance, letrozole, 3C Taxotere/avastin
142	high-grade serous ovarian carcinoma	IIIA2	sensitive	DD paclitaxel/carboplatin/maintenance veliparib
143	high-grade serous ovarian carcinoma	IV	resistant	adjuvant 6C paclitaxel/IP cisplatin; 6C doxil/avastin, avastin/amiridex maintenance, 3C single agent carboplatin, 2C cisplatin/gemcitabine/5FU
147	primary peritoneal high grade serous carcinoma	IVB	sensitive	neoadjuvant DD paclitaxel/carboplatin 4C, adjuvant dd paclitaxel/carboplatin 2C
152	high-grade serous fallopian tube carcinoma	IIIC	sensitive	6C paclitaxel/carboplatin
153	gastric signet adenocarcinoma	IIIB	sensitive	7C fluorouracil/oxaliplatin, 2C fluorouracil alone
157	high-grade serous fallopian tube carcinoma	IVB	sensitive	6C DD carboplatin/paclitaxel
161	primary peritoneal high grade serous carcinoma	IIIC	sensitive	3 cycles neoadjuvant dose-dense carboplatin/taxol, IDS, 3 cycles adjuvant dose-dense carboplatin/taxol
178	uterine carcinosarcoma	IIB	resistant	adjuvant 6C carboplatin/paclitaxel; 2C doxil
182	benign serous cyst, para-ovarian	IIIC	none	none
185	high-grade serous ovarian carcinoma	IIIC	sensitive	6C paclitaxel/carboplatin
186	high-grade serous ovarian carcinoma	IIIC	sensitive	neoadjuvant carboplatin/DD paclitaxel 2C, carboplatin/abraxane C3; adjuvant 2C carboplatin/abraxane, 1C abraxane
189	high-grade serous ovarian carcinoma	IIA	sensitive	neoadjuvant carboplatin/paclitaxel 3C, adjuvant 1C carboplatin/paclitaxel, 2C carboplatin/paclitaxel/bevacizumab, maintenance bevacizumab
198	cervical squamous cell carcinoma	IIIC	sensitive	6C cisplatin with EBRT and brachytherapy
199	high-grade serous ovarian carcinoma	IIIC	sensitive	6C DD carboplatin/taxol/ bevacizumab; bevacizumab maintenance
200	primary peritoneal high grade serous carcinoma	IA	sensitive	neoadjuvant carboplatin/paclitaxel/bevacizumab 3C, a djuvant 6C carboplatin/paclitaxel/bevacizumab, 3C bevacizumab
202	missing from system (same patient as 189)	IIIA		
212	mucinous borderline ovarian tumor	IIIB	none	none
219	serous uterine carcinoma	IVA	chemonaïve	6 C adjuvant carboplatin/paclitaxel
222	high-grade serous fallopian tube carcinoma	IA	sensitive	6C carboplatin/paclitaxel/ bevacizumab/ atezolizumab, olaparib maintenance
225	clear cell ovarian carcinoma			neoadjuvant CT treated
